# Transcriptional response of murine microglia in Alzheimer’s disease and inflammation

**DOI:** 10.1186/s12864-022-08417-8

**Published:** 2022-03-05

**Authors:** Daniel C. Shippy, Jyoti J. Watters, Tyler K. Ulland

**Affiliations:** 1 Department of Pathology and Laboratory Medicine, University of Wisconsin, Madison, WI USA; 2 Department of Comparative Biosciences, University of Wisconsin, Madison, WI USA

**Keywords:** Alzheimer's disease, Microglia, RNA-seq, Microarray, Transcriptomics

## Abstract

**Background:**

Alzheimer’s disease (AD) is a neurodegenerative disorder and is the most common cause of late-onset dementia. Microglia, the primary innate immune cells of the central nervous system (CNS), have a complex role in AD neuropathology. In the initial stages of AD, microglia play a role in limiting pathology by removing amyloid-β (Aβ) by phagocytosis. In contrast, microglia also release pro-inflammatory cytokines and chemokines to promote neuroinflammation and exacerbate AD neuropathology. Therefore, investigating microglial gene networks could identify new targets for therapeutic strategies for AD.

**Results:**

We identified 465 differentially expressed genes (DEG) in 5XFAD versus wild-type mice by microarray, 354 DEG in lipopolysaccharide (LPS)-stimulated N9 microglia versus unstimulated control cells using RNA-sequencing (RNA-seq), with 32 DEG common between both datasets. Analyses of the 32 common DEG uncovered numerous molecular functions and pathways involved in Aβ phagocytosis and neuroinflammation associated with AD. Furthermore, multiplex ELISA confirmed the induction of several cytokines and chemokines in LPS-stimulated microglia.

**Conclusions:**

In summary, AD triggered multiple signaling pathways that regulate numerous genes in microglia, contributing to Aβ phagocytosis and neuroinflammation. Overall, these data identified several regulatory factors and biomarkers in microglia that could be useful in further understanding AD neuropathology.

**Supplementary Information:**

The online version contains supplementary material available at 10.1186/s12864-022-08417-8.

## Background

Alzheimer’s disease (AD) is a neurodegenerative disorder and is the most common cause of late-onset dementia. In the United States, approximately 6.2 million people are living with Alzheimer’s dementia, a number estimated to grow to 13.8 million by 2060 unless medical intervention strategies are developed for AD [1]. AD neuropathology is defined by the aggregation of extracellular amyloid-β (Aβ) plaques followed by the development of intracellular neurofibrillary tangles (NFTs) composed of hyperphosphorylated tau [2]. In addition to Aβ plaques and tau NFTs, neuroinflammation plays a key role in AD neuropathology, promoting numerous inflammatory processes in the central nervous system (CNS) [3].

Microglia, the primary innate immune cells of the CNS, have a complex role in AD neuropathology. In the early stages of AD, microglia reduce Aβ accumulation by phagocytosis, and act as a defense barrier to protect plaque adjacent neurons from neurotoxic effects [4, 5]. Alternatively, microglia can contribute to neuroinflammation by the release of pro-inflammatory cytokines and chemokines, reactive oxygen species, and other molecules associated with increased AD neuropathology [6]. Although the role of microglia in AD is still not entirely understood, it is clear microglia play a key role in the development of AD neuropathology.

Since microglia appear to be an important factor in AD development, investigating microglial gene networks could lead to new therapies to treat AD. Several studies have already highlighted the importance of specific genes involved in microglial metabolism [7] and response to Aβ plaque pathology [8]. In this study, we investigated the transcriptional response of microglia in an AD versus non-AD state using microarray and RNA-sequencing (RNA-seq). We identified 465 differentially expressed genes (DEG) in 5XFAD versus wild-type mice, and 354 DEG in lipopolysaccharide (LPS)-stimulated N9 microglia versus unstimulated control cells, with 32 DEG common to both experiments. Of the 32 DEG, functional enrichment analyses identified numerous processes and pathways in which microglia are potentially involved during AD development. Furthermore, multiplex ELISA confirmed the induction of several cytokines and chemokines in LPS-stimulated microglia that were also differentially expressed in the microarray and RNA-seq datasets. Overall, these data identify novel potential regulatory factors and biomarkers in the microglial response to AD.

## Results

### Transcriptome analyses of microglia in AD

**Fig. 1:**
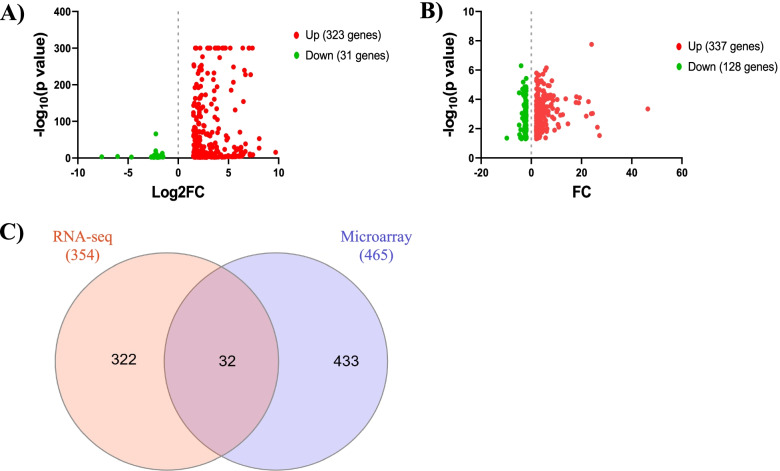
AD transcriptional alterations in microglia. **A** Scatter plot of significantly altered genes (log_2_FC > 1.5, FDR-adjusted *P*-value < 0.05) by RNA-seq in N9 microglia stimulated with LPS (1 µg/ml) for 6 h versus unstimulated control cells. **B** Scatter plot of significantly altered transcripts (FC > 2, FDR-adjusted *P*-value < 0.05) by microarray in microglia isolated from the brains of female 5XFAD mice versus wild-type mice (8 months old). For both scatter plots, up-regulated genes are shown in red and down-regulated genes are shown in green. **C** Venn diagram showing the 354 significantly altered genes in N9 microglia by RNA-seq, 465 significantly altered transcripts in murine microglia by microarray, and 32 significantly altered genes found in both the RNA-seq and microarray data

Transcriptional analysis of LPS-stimulated N9 microglia versus non-stimulated control cells was performed using RNA-seq. A total of 354 significant DEG (log_2_FC > 1.5, FDR-adjusted *P*-value < 0.05) were identified with 323 up-regulated genes and 31 down-regulated genes (Fig. 1A and Additional File 1). Interleukin-1 alpha (*Il1α*) was the most up-regulated gene (log_2_FC = 9.70) and albumin (*Alb*) was the most down-regulated gene (log_2_FC = -7.62) (Table 1).

**Table 1:** Top 10 up-regulated and down-regulated genes in LPS-stimulated N9 microglia versus control cells determined by RNA-seq

Gene	Gene ID^a^	Gene Description	Log_2_FC
*Il1a*	27399	interleukin 1 alpha	9.70
*Ifit3b*	62488	interferon-induced protein with tetratricopeptide repeats 3B	8.08
*Nos2*	20826	nitric oxide synthase 2, inducible	8.07
*Col5a3*	04098	collagen, type V, alpha 3	7.44
*Rsad2*	20641	radical S-adenosyl methionine domain containing 2	7.39
*Gm19410*	09372	predicted gene, 19,410	7.39
*Ifit3*	74896	interferon-induced protein with tetratricopeptide repeats 3	7.21
*Il1f9*	44103	interleukin 1 family, member 9	7.15
*Trim30c*	78616	tripartite motif-containing 30C	7.13
*Ifit1*	34459	interferon-induced protein with tetratricopeptide repeats 1	7.06
*Alb*	29368	albumin	-7.62
*Gc*	35540	vitamin D binding protein	-6.04
*Ttr*	61808	transthyretin	-4.66
*BC021767*	85006	cDNA sequence BC021767	-2.68
*Tlr8*	40522	toll-like receptor 8	-2.65
*4930473A02Rik*	60029	RIKEN cDNA 4930473A02 gene	-2.55
*Fsbp*	94595	fibrinogen silencer binding protein	-2.51
*Nlrp1a*	69830	NLR family, pyrin domain containing 1A	-2.45
*Cgn*	68876	cingulin	-2.41
*Abcd2*	55782	ATP-binding cassette, sub-family D (ALD), member 2	-2.37

^a^All gene IDs start with ENSMUSG000000

We compared this data to publicly available data derived from sorted microglia from female 8-month-old wild-type and 5XFAD mice, a mouse model of AD which accumulates Aβ plaques [9]. Transcriptional analysis of microglia from 5XFAD versus wild-type mice was performed by microarray [10]. A total of 465 significant gene transcripts (FC > 2, FDR-adjusted *P*-value < 0.05) were identified with 337 up-regulated gene transcripts and 128 down-regulated gene transcripts (Fig. 1B and Additional File 2). Glycoprotein (transmembrane) nmb (*Gpnmb*) was the most up-regulated gene transcript (FC = 46.43) and X-linked lymphocyte-regulated 4B (*Xlr4b*) was the most down-regulated gene transcript (FC = -9.76) (Table 2).

**Table 2:** Top 10 up-regulated and down-regulated gene transcripts in 5XFAD versus wild-type mice determined by microarray

Gene	Gene ID^a^	Gene Description	FC
*Gpnmb*	031840	glycoprotein (transmembrane) nmb	46.43
*Ddx3y*	091190	DEAD (Asp-Glu-Ala-Asp) box polypeptide 3, Y-linked	27.16
*Spp1*	112747	secreted phosphoprotein 1	26.34
*Mamdc2*	036069	MAM domain containing 2	24.41
*Cst7*	089200	cystatin F (leukocystatin)	24.03
*Fabp3*	070532	fatty acid binding protein 3, muscle and heart	23.89
*Fabp5*	029046	fatty acid binding protein 5, epidermal	22.76
*Bhlhe40*	032194	basic helix-loop-helix family, member e40	21.89
*Hpse*	045617	heparanase	19.26
*Igf1*	121161	insulin-like growth factor 1	18.88
*Xlr4b*	114506	X-linked lymphocyte-regulated 4B	-9.76
*Gpr165*	033554	G protein-coupled receptor 165	-4.84
*Snord35b*	082833	small nucleolar RNA, C/D box 35B	-4.83
*Xist*	127786	inactive X specific transcripts	-4.70
*4933434E20Rik*	159064	RIKEN cDNA 4933434E20 gene	-4.35
*Snord61*	083176	small nucleolar RNA, C/D box 61	-4.25
*Bank1*	041577	B-cell scaffold protein with ankyrin repeats 1	-4.08
*Ttr*	075312	transthyretin	-3.90
*Fam71a*	171798	family with sequence similarity 71, member A	-3.76
*Il7r*	003981	interleukin 7 receptor	-3.73

^a^ All gene IDs start with ENSMUST00000

In total, 32 genes overlapped between the N9 RNA-seq experiment and 5XFAD microarray dataset (Fig. 1C). Of the 32 total genes, 31 were up-regulated and only one was down-regulated (Table 3). The majority of the most up-regulated genes were cytokines and chemokines involved in inflammation (*Cxcl10*, *Cxcl2*, *Il1β*,* Tnf*). Transthyretin (*Ttr*) was the only down-regulated gene common to both datasets.

**Table 3:** Genes found in both the RNA-seq and microarray experiments

Gene	Gene ID^a^	Gene Description	RNA-seq Log_2_FC	Microarray FC
*Ifit3*	74896	interferon-induced protein with tetratricopeptide repeats 3B	7.21	2.21
*Cxcl10*	34855	chemokine (C-X-C motif) ligand 10	7.04	3.08
*Cxcl2*	58427	chemokine (C-X-C motif) ligand 2	6.60	3.72
*Il1b*	27398	interleukin 1 beta	5.94	7.76
*Oasl2*	29561	2'-5' oligoadenylate synthetase-like 2	5.18	4.29
*Ifit2*	45932	interferon-induced protein with tetratricopeptide repeats 2	4.58	3.15
*Ifi204*	73489	interferon activated gene 204	4.13	2.70
*Tnf*	24401	tumor necrosis factor	3.87	2.41
*Slfn5*	54404	schlafen 5	3.67	3.57
*Prdm1*	38151	PR domain containing 1, with ZNF domain	3.29	2.57
*Slfn2*	72620	schlafen 2	3.16	2.25
*Il1rn*	26981	interleukin 1 receptor antagonist	2.99	4.35
*Cd83*	15396	CD83 antigen	2.97	2.17
*Gvin1*	45868	GTPase, very large interferon inducible 1	2.82	2.20
*Phlda1*	20205	pleckstrin homology like domain, family A, member 1	2.75	3.85
*Gm1966*	73902	predicted gene 1966	2.44	2.19
*Gpr84*	63234	G protein-coupled receptor 84	2.39	2.02
*Cd69*	30156	CD69 antigen	2.36	9.28
*Slc7a11*	27737	solute carrier family 7 (cationic amino acid transporter, y + system), member 11	2.31	2.59
*Cd274*	16496	CD274 antigen	2.09	5.72
*Irak3*	20227	interleukin-1 receptor-associated kinase 3	2.06	2.20
*Serpine1*	37411	serine (or cysteine) peptidase inhibitor, clade E, member 1	2.03	4.16
*Mmp12*	49723	matrix metallopeptidase 12	1.77	2.73
*Tlr2*	27995	toll-like receptor 2	1.77	2.70
*Olr1*	30162	oxidized low density lipoprotein (lectin-like) receptor 1	1.75	2.70
*Rab11fip1*	31488	RAB11 family interacting protein 1 (class I)	1.66	2.42
*Cd300lf*	47798	CD300 molecule like family member F	1.63	3.45
*C3*	24164	complement component 3	1.59	2.12
*Itga5*	00555	integrin alpha 5 (fibronectin receptor alpha)	1.57	4.85
*Bcl2a1d*	99974	B cell leukemia/lymphoma 2 related protein A1d	1.53	4.05
*Plaur*	46223	plasminogen activator, urokinase receptor	1.51	3.60
*Ttr*	61808	transthyretin	-4.66	-3.9

^a^All gene IDs start with ENSMUSG000000

### Pathway and functional prediction of microglia in AD

**Fig. 2:**
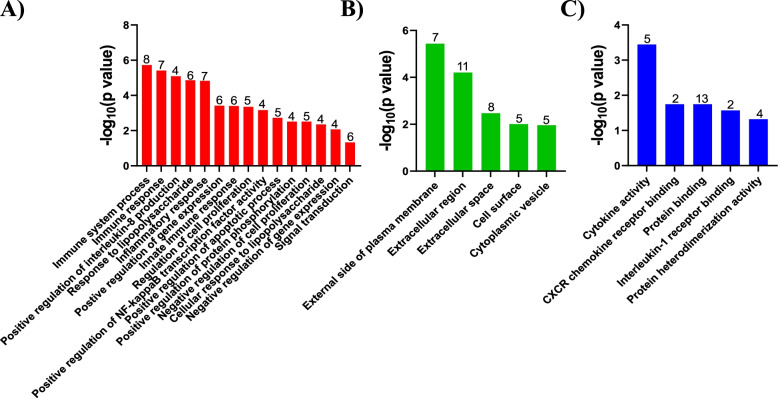
GO enrichment analysis. Biological function analyses for the 32 genes found in both the RNA-seq and microarray experiments was performed using DAVID. Analyses were performed for Biological Process (BP) (**A**), Cellular Component (CC) (**B**), and Molecular Function (MF) (**C**). Pathways are shown in descending order based on –log_10 _*P*-values. The number of genes associated with each GO term is shown above each bar. Only GO terms with a *P*-value < 0.05 are shown

Geno ontology (GO), Kyoto Encyclopedia of Genes and Genomes (KEGG), and Search Tool for the Retrieval of Interacting Genes/Proteins (STRING) analyses were performed on the 32 genes found in both the RNA-seq and microarray datasets. Biological Process (BP) GO indicated the DEG participated in immune system process (*Oasl2*, *Cd300lf*, *Prdm1*, *C3*,* Ifit2*, *Ifit3*, *Olr1*, *Tlr2*), immune response (*Oasl2*, *Cd274*, *Cxcl10*,* Cxcl2*,* Il1β*, *Tlr2*, *Tnf*), positive regulation of interleukin-8 (*Il1β*, *Serpine1*, *Tlr2*, *Tnf*), response to LPS (*Cxcl10*,* Cxcl2*,* Il1β*,* Irak3*, *Tlr2*, *Tnf*), inflammatory response (*Cxcl10*, *Cxcl2*, *C3*, *Il1β*, *Olr1*,* Tlr2*,* Tnf*), positive regulation of gene expression (*Prdm1*, *Il1β*,* Plaur*,* Serpine1*,* Tlr2*,* Tnf*), innate immune response (*Oasl2*,* Prdm1*,* C3*, *Ifit2*, *Ifit3*, *Tlr2*), regulation of cell proliferation (*Prdm1*,* Cxcl10*, *Cxcl2*, *Serpine1*,* Tnf*), positive regulation of NF-kappaB transcription factor activity (*Il1β*,* Irak3*,* Tlr2*, *Tnf*), positive regulation of apoptotic process (*Bcl2a1d*,* Ifit2*, *Il1β*,* Phlda1*,* Tnf*), positive regulation of protein phosphorylation (*C3*, *Il1β*, *Plaur*, *Tnf*), negative regulation of cell proliferation (*Ifit3*, *Il1β*,* Slfn2*,* Tlr2*,* Tnf*), cellular response to LPS (*Cxcl10*,* Cxcl2*, *Serpine1*, *Tnf*), negative regulation of gene expression (*Prdm1*,* Il1β*,* Serpine1*, *Tnf*), and signal transduction (*Cd274*,* Cd83*,* Gpr84*,* Irak3*,* Tlr2*,* Tnf*) (Fig. 2A). Cellular Component (CC) GO indicated the DEG were located in the external side of plasma membrane (*Cd274*, *Cd69*, *Cd83*, *Cxcl10*, *Itga5*, *Tlr2*, *Tnf*), extracellular region (*Cxcl10*, *Cxcl2*, *C3*, *Il1β*, *Il1rn*, *Mmp12*, *Olr1*, *Plaur*, *Serpine1*, *Ttr*, *Tnf*), extracellular space (*Cxcl10*, *Cxcl2*, *C3*, *Il1β*, *Il1rn*, *Serpine1*, *Ttr*, *Tnf*), cell surface (*Cd274*, *Itga5*, *Slc7a11*, *Tlr2*, *Tnf*), and cytoplasmic vesicle (*Rab11fip1*, *Itga5*, *Il1β*, *Phlda1*, *Tlr2*) (Fig. 2B). Molecular Function (MF) GO indicated the DEG were involved in cytokine activity (*Cxcl10*, *Cxcl2*, *Il1β*, *Il1rn*, *Tnf*), CXCR chemokine receptor binding (*Cxcl10*, *Cxcl2*), protein binding (*Cd274*, *Cd300lf*, *Prdm1*, *C3, Itga5*, *Ifi204*, *Ifit3*, *Irak3*, *Plaur*, *Serpine1*, *Tlr2*, *Ttr*, *Tnf*), interleukin-1 receptor binding (*Il1β*, *Il1rn*), and protein heterodimerization activity (*Bcl2a1d*, *Irak3*, *Tlr2*, *Ttr*) (Fig. 2C).

**Fig. 3:**
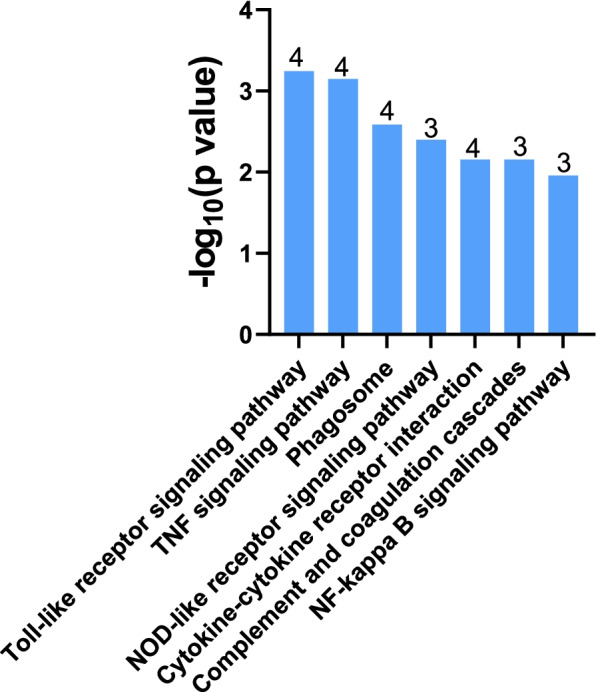
KEGG pathway enrichment analysis. KEGG pathway analysis was performed on the 32 genes found in both the RNA-seq and microarray data using DAVID. Pathways are shown in descending order based on –log_10 _*P*-values. The number of genes associated with each pathway is shown above each bar. Only pathways with a *P*-value < 0.05 are shown

KEGG analysis was performed on the 32 common genes found in both the microarray and RNA-seq datasets to identify pathways associated with AD. KEGG identified a total of seven pathways (*P* < 0.05) associated with the 32 genes (Fig. 3). The pathways included toll-like receptor signaling (*Tlr2*, *Tnf*, *Il1β*, *Cxcl10*), TNF signaling (*Cxcl10*, *Cxcl2*, *Il1β*, *Tnf*), phagosome (*C3*, *Itga5*, *Olr1*, *Tlr2*), NOD-like receptor signaling (*Cxcl2*, *Il1β*, *Tnf*), cytokine-cytokine receptor interaction (*Cxcl10*, *Cxcl2*, *Il1β*, *Tnf*), complement and coagulation cascades (*C3*, *Plaur*, *Serpine1*), and NF-kappa B signaling (*Bcl2ald*, *Il1β*, *Tnf*).

**Fig. 4:**
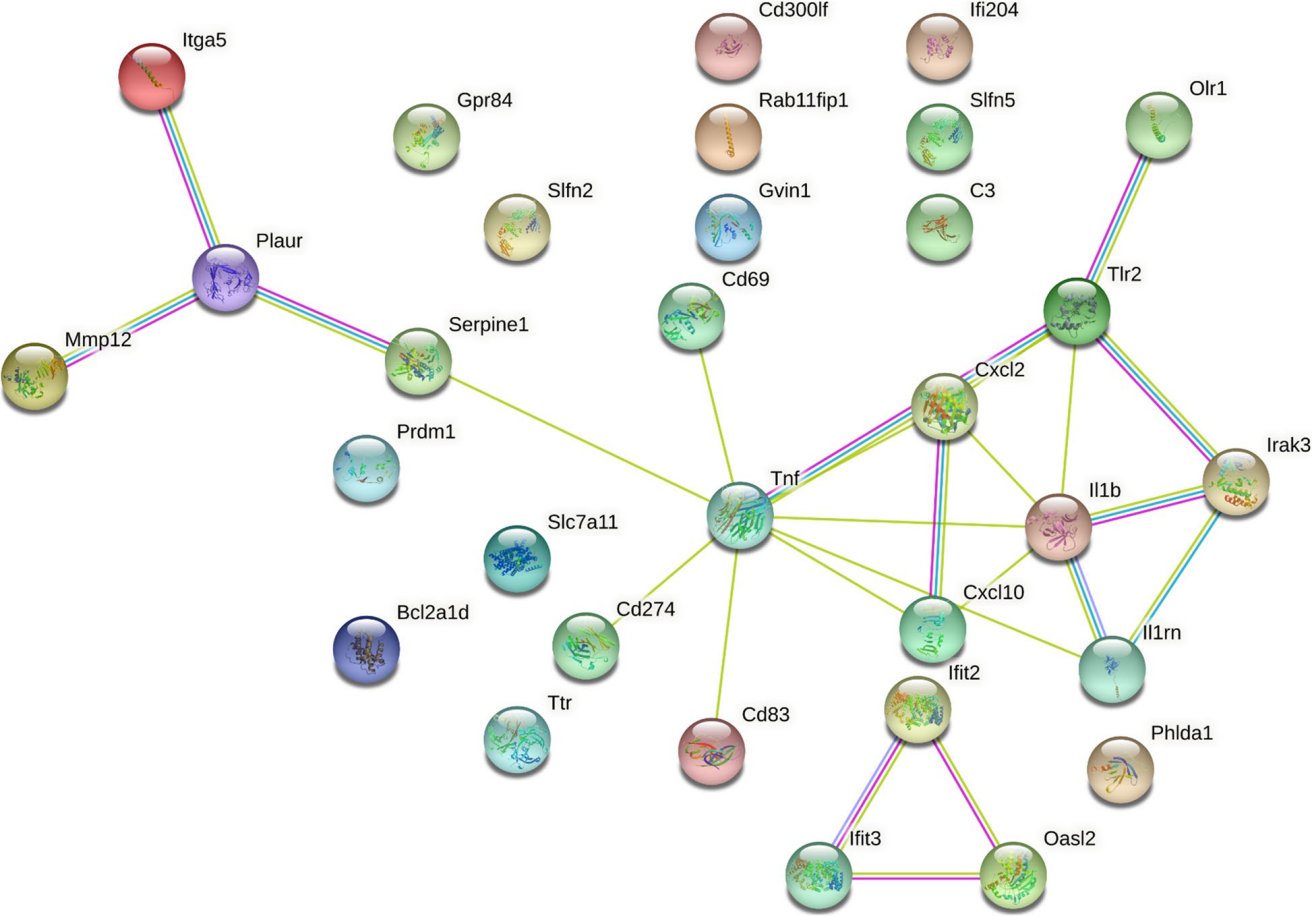
PPI analysis using STRING. STRING analysis was performed on the 32 genes found in both the RNA-seq and microarray data. For the analysis, text mining, experiments, and databases were chosen for active interaction sources, and a high value of 0.700 was selected as the minimum required interaction score. Line colors represent known interactions from curated databases (blue), experimentation (purple) and text mining (yellow)

To further understand the interactions of the DEG, we performed protein–protein interaction (PPI) analysis on the 32 genes found in both the RNA-seq and microarray datasets using STRING. For the analysis, text mining, experiments, and databases were chosen for the interaction sources, and the high confidence value of 0.700 was selected as the minimum required interaction score threshold. Of the proteins encoded by the 32 identified DEG, two distinct networks emerged with 15 proteins in one network (IL1β, Irak3, Il1rn, Cxcl2, Cxcl10, Tnf, Tlr2, Olr1, Cd83, Cd69, Cd274, Serpine1, Plaur, Itga5, Mmp12), three proteins in the second network (Ifit2, Ifit2, Oasl2), and 13 of the proteins not clustering (Fig. 4). The database did not recognize predicted gene 1966 (*Gm1966*). The results suggest that the given proteins were highly enriched (*P* < 1 × 10^–16^).

### Secretion of cytokines and chemokines by microglia

**Fig. 5:**
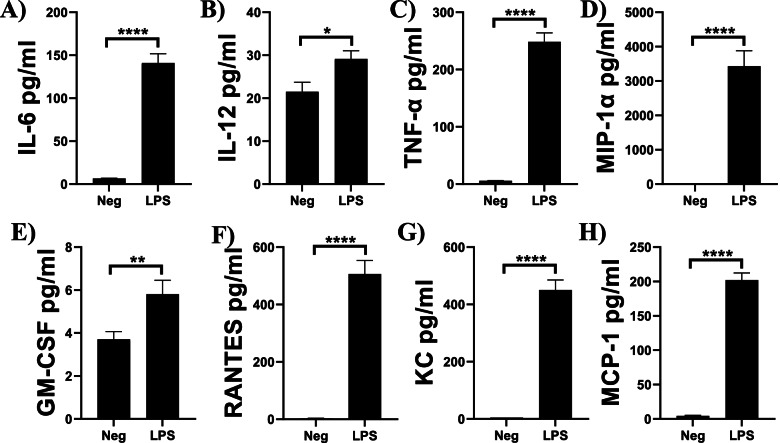
Cytokines and chemokines induced by LPS in primary microglia. Primary microglia were stimulated with LPS (50 ng/ml) for 6 h. Supernatants were collected and assayed for 22 cytokines and chemokines using a multiplex assay. Experiments were performed in triplicate. Results are from three independent experiments. Data are shown as mean ± SEM. ****P ≤ 0.0001, **P ≤ 0.01, *P ≤ 0.05

**Fig. 6:**
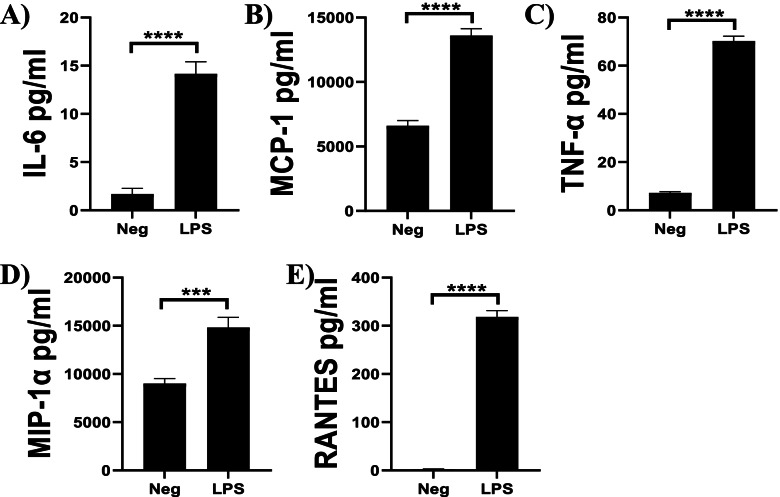
Cytokines and chemokines secreted by N9 microglia following LPS stimulation. N9 microglia were stimulated with LPS (1 µg/ml) for 6 h. Supernatants were collected and assayed for 22 cytokines and chemokines using a multiplex assay. Experiments were performed in triplicate. Results are from three independent experiments. Data are shown as mean ± SEM. ****P ≤ 0.0001, ***P ≤ 0.001

Since so many DEG were cytokines and chemokines associated with inflammation, we performed multiplex ELISAs (22-plex) on supernatants from LPS-stimulated bone marrow-derived macrophages (BMDM), primary microglia, and N9 microglia. In BMDM, IL-α, IL-1β, IL-2, IL-5, IL-6, IL-10, IL-12, MCP-1, IFN-γ, TNF-α, MIP-1α, GM-CSF, RANTES, KC, MDC, TARC, and TCA-3 showed significant induction when stimulated with LPS versus non-stimulated control cells (Additional File 3). In primary microglia, IL-6, IL-12, TNF-α, MIP-1α, GM-CSF, RANTES, KC, and MCP-1 all showed significant induction in LPS-stimulated primary microglia versus non-stimulated control cells (Fig. 5). In N9 microglia, IL-6, MCP-1, TNF-α, MIP-1α, and RANTES all showed significant induction in LPS-stimulated N9 microglia versus non-stimulated control cells (Fig. 6). Cytokines and chemokines from BMDM, primary microglia, and N9 microglia that showed no significant difference between LPS-stimulated and control cells are shown in Additional File 4.

## Discussion

A thorough understanding of the molecular characteristics and regulatory pathways of microglia is essential to develop therapeutic strategies for AD. Accordingly, numerous studies have performed transcriptome analyses to elucidate the role of specific genes in microglia important in AD neuropathology, including Trem2 and ApoE [7, 10–13]. Other studies have investigated the transcriptome of microglia under distinct biological activities, such as Aβ plaque phagocytosis [14], tau pathology [15] and metabolism [16]. In this study, we used a two-pronged approach to determine DEG in AD and inflammation. First, we determined the DEG in microglia from 5XFAD mice versus wild-type mice by microarray. We then determined the DEG in LPS-stimulated microglia using RNA-seq. Altogether, 32 DEG overlapped between the two datasets.

Neuroinflammation by microglia is hypothesized to exacerbate AD neuropathology [17]. In our study, several immunological pathways associated with inflammation were identified in AD-associated microglia that correlated with DEG of well-known pro-inflammatory cytokines and chemokines, such as IL-1β, TNF, CXCL10, and CXCL2. Caspase-1 dependent IL-1β secretion occurs during NLRP3 inflammasome activation, and activation of the inflammasome has emerged as an important mechanism in chronic neuroinflammation in AD [18]. Furthermore, immunotherapies blocking inflammasome activation are being investigated for use in AD and other inflammatory diseases [19–21]. BP GO analysis also identified *Olr1* as an inflammatory and immune system response gene. Several studies suggest a role for *Olr1* in lipid metabolism, and genetic variation in *Olr1* as a risk factor for AD [22–24], but little is known about *Olr1* in AD-associated neuroinflammation. Furthermore, *Olr1* was recognized as a gene involved in the phagosome pathway by KEGG, suggesting an important role in multiple AD pathogenic processes.

Genes not previously characterized in AD were identified by KEGG to be involved in molecular signaling pathways important in AD development. The B cell leukemia/lymphoma 2 related protein A1d (*Bcl2a1d*) was identified as being involved in the nuclear factor kappa-light-chain-enhancer of activated B cells (NF-κB) signaling pathway. Activation of NF-κB is an important mechanism in chronic neuroinflammation that significantly increases AD pathology [25]. Furthermore, the plasminogen activator, urokinase receptor (*Plaur*) was recognized as being associated with complement and coagulation cascades. Complement and coagulation cascades are associated with blood–brain barrier dysfunction and AD progression in mice [26] and humans [27]. Characterization of these genes in the context of AD could further our understanding of the underlying regulatory mechanisms in AD pathogenesis.

Biomarkers can be a useful tool to predict and diagnosis a variety of neurological disorders, including AD [28]. In our study, the only down-regulated gene amongst the 32 DEG was transthyretin (*Ttr*). Indeed, Ttr has been implicated in numerous processes associated with AD, including Aβ binding [29], Aβ transport at the blood–brain barrier (BBB) [30], toxicity [31, 32], and neuroprotection by interfering with Aβ formation [29, 33, 34]. Ttr has been previously identified as being decreased in the cerebral spinal fluid (CSF) [35] and blood [36–38] of AD patients, and is being investigated as a blood biomarker for AD [38, 39]. Several other DEG in our study could also potentially be used as biomarkers due to their high-level of differential expression between groups (greater than threefold in both assays), including *Ifit3*, *Cxcl10*, *Cxcl2, Il1β*, *Oasl2*, *Ifit2*, and *Slfn5*. Further investigation will be needed, as clinically useful biomarkers for AD should be inexpensive, non-invasive, reliably detectable and able to distinguish AD from other forms of dementia [40].

There are several limitations of our study which are common to most studies directed at identifying dynamic alterations in gene expression networks in AD. Microgliosis is a hallmark of AD neuropathology, resulting in large numbers of microglia concentrated around Aβ plaques [41]. Since the role of microglia in AD is not fully understood, it is difficult to ascertain whether the altered genes identified in our study are involved in disease progression or in the neuroprotective microglia response. Another caveat to our study is that murine microglia were used in both experiments. Although the value of murine microglial research in AD cannot be overstated, the transcriptional signature of microglia in human AD is drastically different than that of murine microglia [11, 42]. When comparing human and mouse AD models, transgenic mice overexpress Aβ in a non-physiological manner, resulting in rapid Aβ accumulation and a higher plaque burden when compared to human AD [43, 44]. Although the 5XFAD mouse model does not perfectly recapitulate human disease studies, comparing the microglial response in humans and mice have been mixed, with some indicating that the response may be different [11, 42] and some studies indicating that the microglial response in both humans and mice may be similar [7, 45]. This is likely due to differences in the complexity of human disease where both plaques and neurofibrillary tangles are present compared to the 5XFAD mice in which only plaques form. Therefore, in vitro and in vivo models of AD remain powerful tools to begin to dissect underlying microglial gene regulatory mechanisms in early AD pathology.

## Conclusions

In summary, we identified 465 DEG in 5XFAD versus wild-type mice by microarray, 354 DEG in LPS-stimulated N9 microglia versus unstimulated control cells using RNA-seq, with 32 DEG common between both data sets. Analyses of the 32 DEG uncovered numerous molecular functions and pathways involved in Aβ phagocytosis and neuroinflammation associated with AD that may be further investigated. Furthermore, multiplex ELISA confirmed the induction of several cytokines and chemokines in LPS-stimulated microglia. Overall, these data identified several regulatory factors and biomarkers in microglia that could be useful in further understanding AD neuropathology.

## Methods

### Ethical approval and consent to participate

All methods were carried out in accordance with relevant local and University of Wisconsin guidelines and regulations. All animals were handled in accordance with the Animal Research: Reporting of in vivo Experiments (ARRIVE) guidelines and the University of Wisconsin’s Institutional Animal Care and Use Committee policies and our approved protocols.

### Cell culture assays

BMDM were prepared as previously described [19]. Briefly, tibias were removed from wild-type C57BL/6J mice (6–10 months old) (The Jackson Laboratory), flushed with complete RPMI media supplemented with 20% L-cell conditioned media (LCCM) [19], and cultured for 4–7 days prior to use. Differentiated BMDM were seeded at a cell density of 400,000 cells/well in a 24-well tissue culture plate. Cells were stimulated with ultrapure LPS (50 ng/ml) from *Escherichia coli* O111:B4 (InvivoGen) for 6 h. Supernatants were flash frozen on dry ice and stored at -80℃ until use.

Microglia were prepared from wild-type C57BL/6J mice (5–8 months old) (The Jackson Laboratory) as previously described [7]. Microglia were isolated using Magnetic Activated Cell Sorting (MACS, Miltenyi Biotec) according to manufacturer’s instructions. Briefly, mice were perfused with cold PBS containing 0.1% heparin. Brains were collected in C-tubes (Miltenyi Biotec, Cat. No. 130–096-334) and dissociated using a Neural Tissue Dissociation Kit (T) (Miltenyi Biotec, Cat. No. 130–093-231). Cell suspensions were filtered through a 70 µM cell strainer (Corning Falcon™, Cat. No. 352350). Microglia were labeled with anti-mouse CD45 magnetic beads (Miltenyi Biotec, Cat. No. 130–052-301) and isolated on LS columns (Miltenyi Biotec, Cat. No. 130–042-401). LS columns were washed three times with MACS buffer (PBS containing 0.5% BSA and 1 mM EDTA) before elution. This method allows for preparation of microglial cultures of high purity (> 95%). Purity of isolated microglia are routinely confirmed by flow cytometry. Approximately 50,000 microglia/well were seeded on poly-L-lysine coated 24-well plates in complete RPMI media supplemented with 20% LCCM [19] and human TGF-β (10 ng/ml) (PeproTech, Cat. No. 100–21). Media was changed three days after plating, and cells were used in the week following the media change. Microglia were stimulated with LPS (50 ng/ml) for 6 h. Supernatants were flash frozen on dry ice and stored at -80℃ until use.

Immortalized murine N9 microglia were cultured as previously described [46]. N9 microglia were seeded at a cell density of 250,000 cells/well in a 24-well tissue culture plate. Cells were stimulated with LPS (1 µg/ml) [47] for 6 h. Supernatants were flash frozen on dry ice and stored at -80℃ until use.

### ELISA

BMDM, primary microglia, and N9 microglial supernatants were assayed for IL-1α, IL-1β, IL-2, IL-3, IL-4, IL-5, IL-6, IL-10, IL-12, IL-13, IL-17, MCP-1, IFN-γ, TNF-α, MIP-1α, RANTES, GM-CSF, Eotaxin, KC, MDC, TARC, and TCA-3 using a multiplex assay (Quansys Biosciences).

### RNA-seq

RNA was extracted from immortalized N9 microglia stimulated with LPS (1 µg/ml) [47] for 6 h using a RNeasy Plus Mini Kit (Qiagen, Cat. No. 74134). The N9 microglial cell line is derived from mouse brain and shares numerous phenotypic traits with primary mouse microglia [48]. Quality and quantity of RNA was assessed using an Agilent 2100 Bioanalyzer (Agilent Technologies) and a Nanodrop spectrophotometer (Thermo Scientific). All samples had an RNA integrity number (RIN) of 9.7 or higher. RNA library preparation and transcriptome sequencing were performed by Novogene using the Illumina NovaSeq 6000 Sequencing System. Bioinformatics analysis was performed by Novogene with differential expression analysis performed using the DESeq2 R package (1.20.0). The resulting *P*-values were adjusted using the Benjamini and Hochberg method for controlling the false discovery rate [49]. Genes with FDR-adjusted *P*-values < 0.05 and log2FC > 1.5 were considered differentially expressed.

### Microarray

The microarray has been published in a previous study [10] and the publicly available dataset was used (GSE65067). Briefly, microglia from female 8 month old wild-type (*n* = 3) and 5XFAD (*n* = 5) mice (The Jackson Laboratory) were FACS-sorted directly into RTL-plus lysis buffer. RNA extraction from microglia was performed using a RNeasy Plus Micro Kit (Qiagen, Cat. No. 74034). Microarray hybridization (Affymetrix MoGene 1.0 ST array) and data processing were performed at the Washington University Genome Center. Raw data were normalized using the Robust Multi-Array (RMA) method and genes were pre-filtered for expression value greater than or equal to 120 expression units. This method provides a cut-off above which genes have a 95% chance of expression demonstrated in Immgen dataset, which uses the same array platform [10]. *P*-values were calculated using a Student’s t-test and FDR-adjusted *P*-values were calculated using the Benjamini and Hochberg method [49]. Transcripts with FDR-adjusted *P*-values < 0.05 and FC > 2 were considered differentially expressed.

### Functional enrichment analyses

Genes found to be differentially expressed in both the RNA-seq and microarray experiments were selected for biological function and pathway analysis. The gene list was uploaded into the Database for Annotation, Visualization and Integrated Discovery (DAVID, v. 6.8) [50, 51] for GO and KEGG pathway analysis. GO gene count thresholds of 4, 2, and 2 were used for BP, CC, and MF respectively. Each GO and KEGG pathway with a *P*-value < 0.05 was considered significant. Additionally, PPI analysis was performed to identify interactions of the selected proteins based on their gene IDs using the STRING database [52]. For the analysis, text mining, experiments, and databases were chosen for active interaction sources using the high confidence (0.700) threshold setting.

### Statistical analyses

Statistical analysis was performed using Prism 9.0.0 (GraphPad). Data are presented as mean ± SEM. Comparison between two groups was performed using a Student’s *t*-test. A *P*-value ≤ 0.05 (*P ≤ 0.05, **P ≤ 0.01, ***P ≤ 0.001 and ****P ≤ 0.0001) was used as the significance cutoff. The Venn diagram demonstrating overlap in DEG amongst the two datasets was generated using InteractiVenn [53].

## Supplementary Information

**Additional file1.**

**Additional file2.**

**Additional file3.**

**Additional file 4.**

## Data Availability

The datasets generated and/or analyzed during the current study are available in the Gene Expression Omnibus (GEO) repository, GSE65067 and GSE183038.
