# Supplementary material for: Transcriptional response of murine microglia in Alzheimer’s disease and inflammation
Source: BMC Genomics. 2022 Mar 5;23:183. doi: 10.1186/s12864-022-08417-8 (PMC8898509; doi:10.1186/s12864-022-08417-8)
Supplement: Supplementary file 3 — Additional file3. [file 12864_2022_8417_MOESM3_ESM.pptx]

## Slide 1
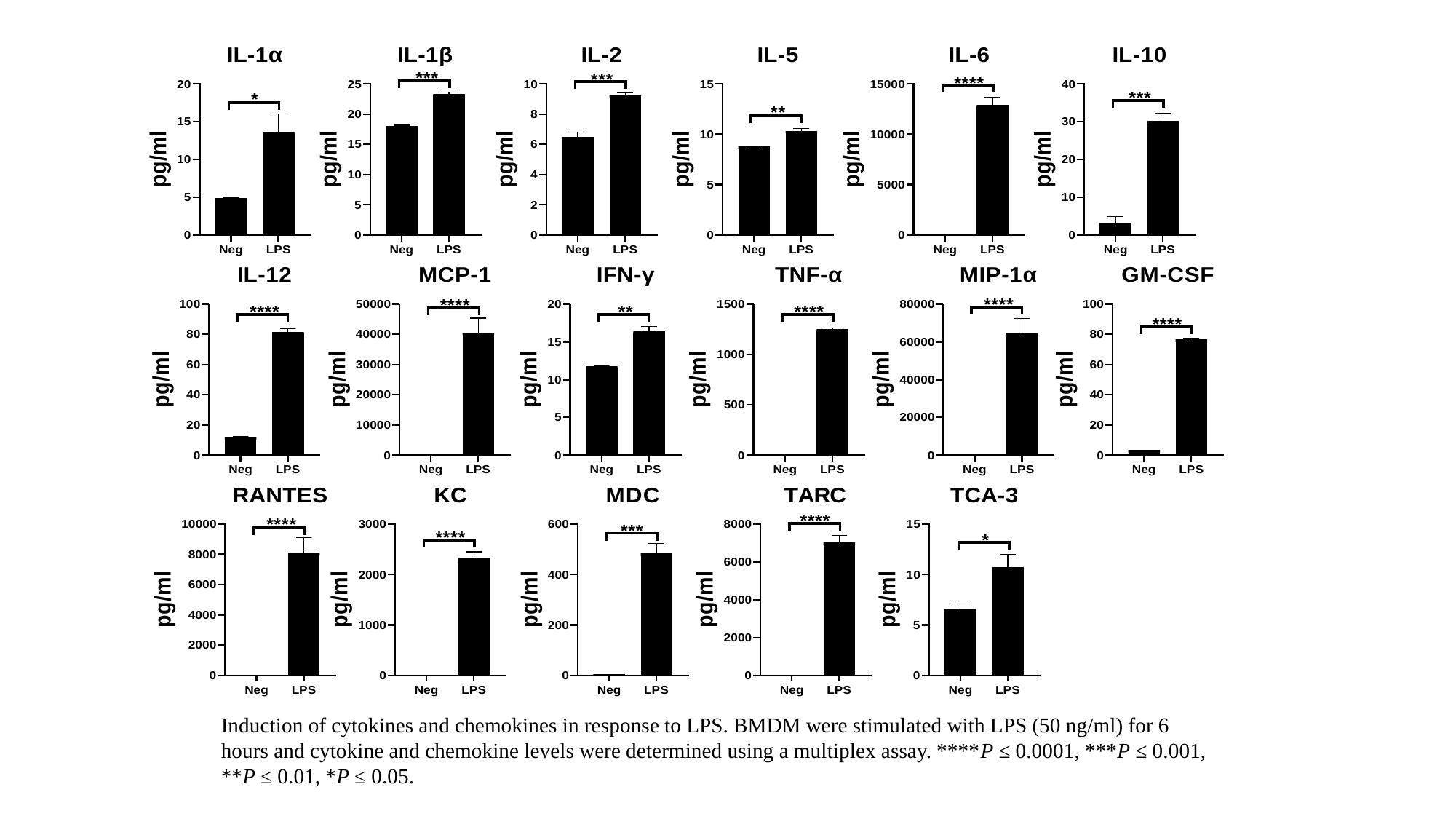

Induction of cytokines and chemokines in response to LPS. BMDM were stimulated with LPS (50 ng/ml) for 6 hours and cytokine and chemokine levels were determined using a multiplex assay. ****P ≤ 0.0001, ***P ≤ 0.001, **P ≤ 0.01, *P ≤ 0.05.
