# Supplementary material for: Transcriptional response of murine microglia in Alzheimer’s disease and inflammation
Source: BMC Genomics. 2022 Mar 5;23:183. doi: 10.1186/s12864-022-08417-8 (PMC8898509; doi:10.1186/s12864-022-08417-8)
Supplement: Supplementary file 4 — Additional file 4. [file 12864_2022_8417_MOESM4_ESM.pptx]

## Slide 1
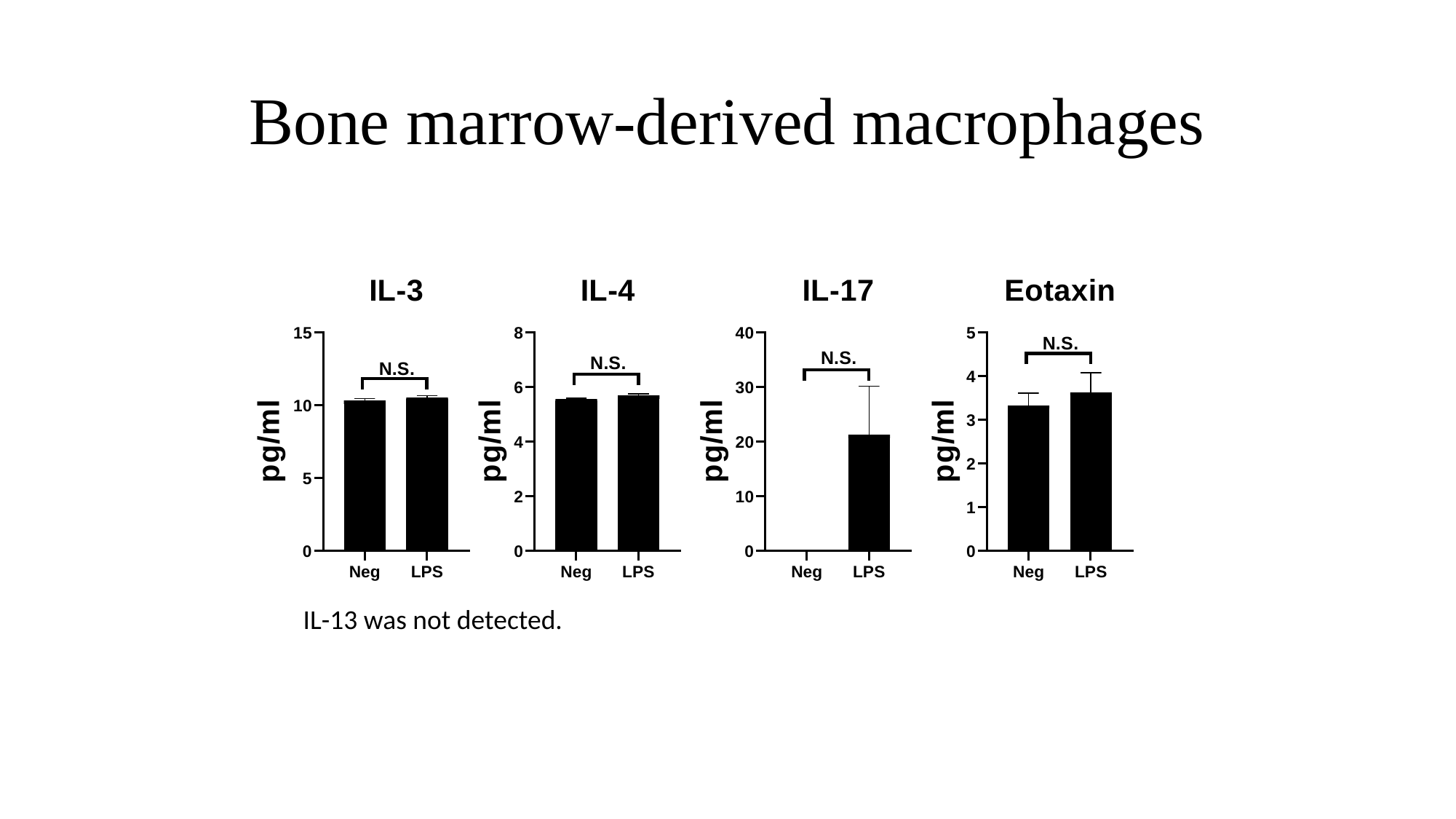

# Bone marrow-derived macrophages
IL-13 was not detected.

## Slide 2
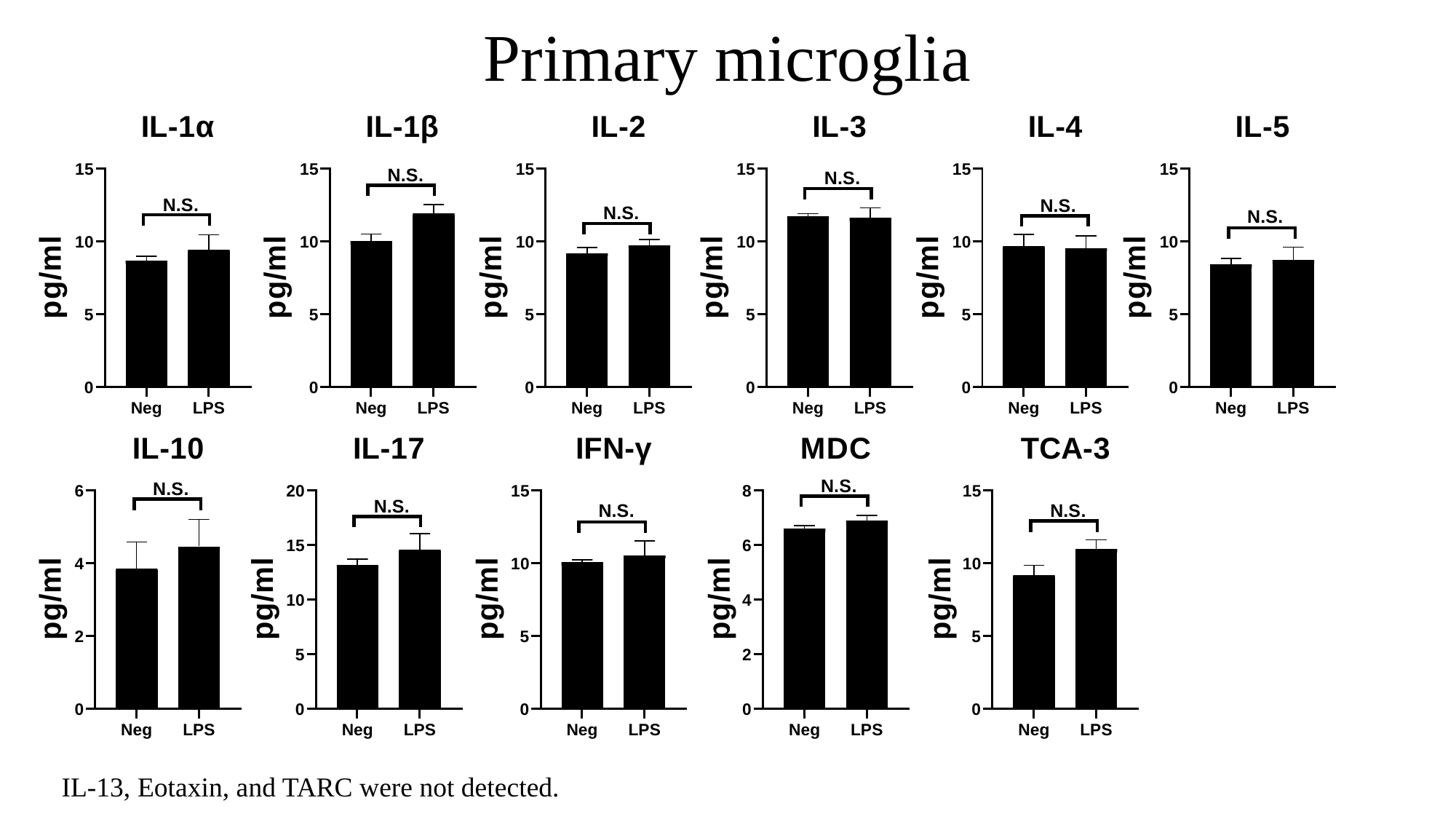

# Primary microglia
IL-13, Eotaxin, and TARC were not detected.

## Slide 3
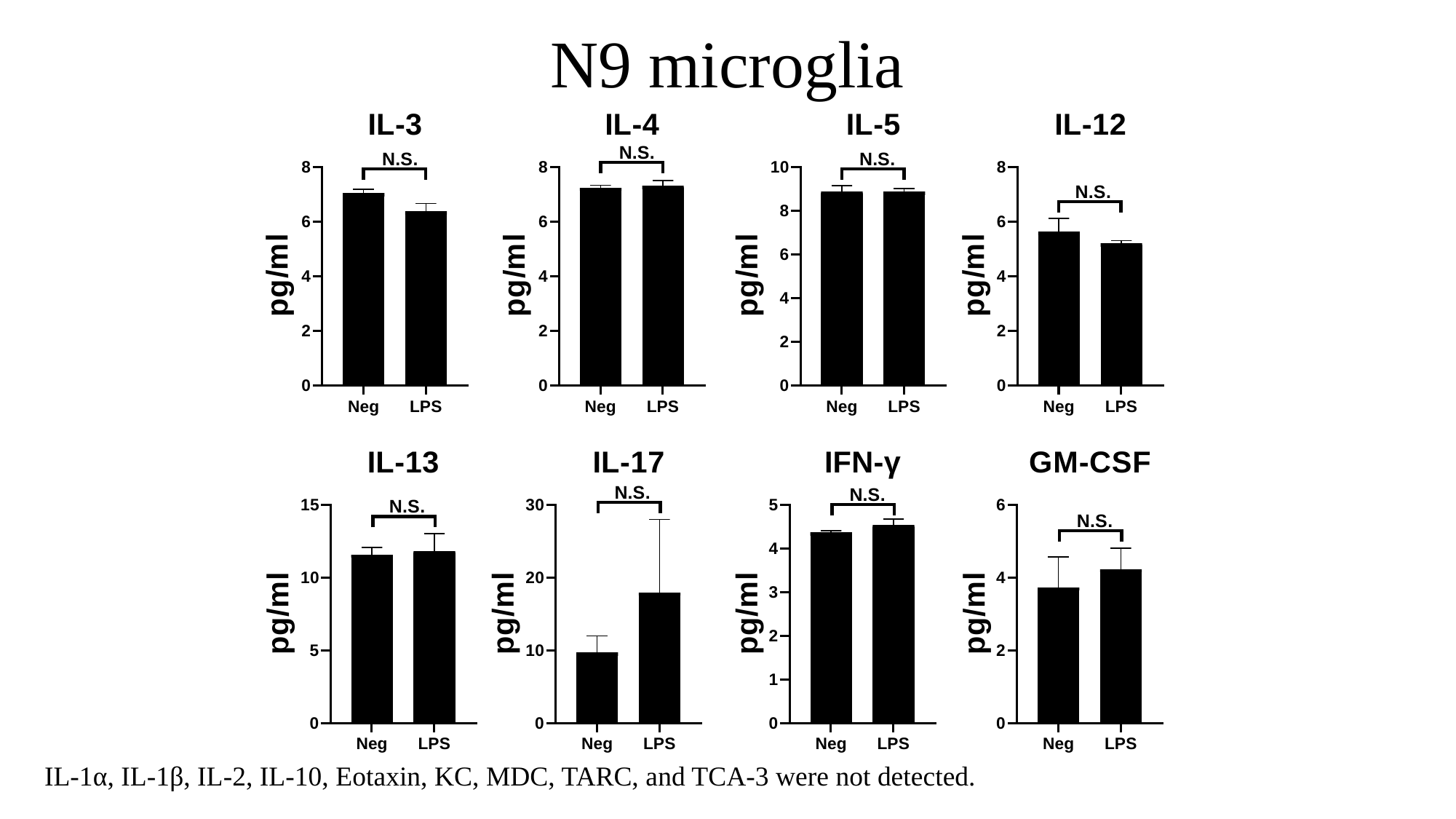

# N9 microglia
IL-1α, IL-1β, IL-2, IL-10, Eotaxin, KC, MDC, TARC, and TCA-3 were not detected.
